# High-Metastatic Melanoma Cells Promote the Metastatic Capability of Low-Metastatic Melanoma Cells *via* Exosomal Transfer of miR-411-5p

**DOI:** 10.3389/fonc.2022.895164

**Published:** 2022-05-20

**Authors:** Hao Chen, Bin Zeng, Xiaoshuang Li, Qiting Zhao, Doudou Liu, Yuting Chen, Yuhan Zhang, Jianyu Wang, H. Rosie Xing

**Affiliations:** ^1^ State Key Laboratory of Ultrasound in Medicine and Engineering, College of Biomedical Engineering, Chongqing Medical University, Chongqing, China; ^2^ Chongqing Key Laboratory of Biomedical Engineering, Chongqing Medical University, Chongqing, China; ^3^ Institute of Life Sciences, Chongqing Medical University, Chongqing, China

**Keywords:** exosome, metastatic microenvironment, melanoma, miR-411-5p, mitogen-activated protein kinase (MAPK) signal pathway

## Abstract

Melanoma is characterized by high rate of metastasis and mortality. Effective management of metastatic melanoma depends on renewed mechanistic understanding underlying melanoma progression and metastasis. The role of exosomes in mediating the interactions between cancer cells and the metastatic microenvironment is at the forefront of cancer research. Previous researches on the function of exosomes in metastasis have been primarily focused on tumor cell-derived exosomes in modifying the biological functions of stromal cells. Whether the cancer cells at the involved organ can modify the metastatic capability of each other has not been demonstrated. In this study, a paired M14 melanoma derivative cell line, i.e., M14-OL and POL, that we established and characterized were employed. Oligo-metastatic (M14-OL) and poly-metastatic (M14-POL) cell line were generated from three consecutive rounds of *in vivo* selection and passage. They exhibit high (POL cells) and low (OL cells) metastatic colonization efficiency *in vivo*, respectively. We show that exosomal crosstalk between metastatic cancer cells is a new mechanism of cancer metastasis. High-metastatic melanoma cells (POL) can augment the metastatic colonization capability of the low-metastatic melanoma cells (OL). POL achieves this goal by utilizing its exosomes to deliver functional miRNAs, in this case, miR-411-5p, to the OL cell. Upon entering OL cells, exosomal miR-411-5p enhance metastatic colonization efficiency by activation of the ERK signaling pathway. Moreover, miR-411-5p expression is higher in cancer tissues of other cancer types (colon, lung, rectum) compared with that of respective normal tissues. The clinical relevance of the present finding merits future investigations.

## Introduction

Melanoma, originated from melanocytes, is characterized by high aggressiveness, metastasis and poor prognosis (1). In the past 50 years, the incidence of melanoma has been increasing steadily at 3%-7%, becoming one of the fastest annual growth rates of all malignancies (2, 3). Although metastatic melanoma patients may benefit from targeted and immunotherapies, the mean survival remains less than 1 year (4). While the high mortality rate of metastatic melanoma is related to effective metastatic colonization of the distant organs, particularly the lung, the underlying mechanisms remain incomplete.

The efficiency of tumor cells metastasis is mainly limited by the stage of proliferative colonization (5–7). A new microenvironment for tumor growth needs to be established at the stage of colonization and the process is complex and multifactorial. Communications between cancer cells and tumor microenvironment during metastasis, are achieved by direct cell-cell interactions, or by indirect paracrine interactions (8).

Exosomes are nanosized vesicles with a diameter of 30-150nm. Exosomes are secreted by many types of cells and can be detected in bodily fluids including saliva, blood and urine (9, 10). Exosomes are a new class of mediators of cell–cell communication. They carry a variety of biologically active materials including miRNA, mRNA, DNA and proteins and can be transferred from donor cells to the target cells (11). miRNAs are 19-22 nt non-coding RNA, which has been identified in exosomes (12, 13). Recent research on exosomes show that exosomes secreted by tumor cells may play an important role in the establishment of pre-metastatic niche and in metastasis (14–17). Previous studies on the role of exosomes in metastasis have been mainly focused on tumor cell-derived exosomes in regulating the functions of stromal cells at the distant organ (18–20). However, there are few studies on the role of exosomes secreted by tumor cells in modifying the metastatic behavior of other tumor cells which in turn impact oncogenesis and metastatic progression. Whether the cancer cells at the involved organ can change the metastatic capability of each other, a novel mechanism of metastasis, has not been demonstrated before this study.

In this study, a paired M14 melanoma derivative cell line, i.e., M14-OL and POL, that we established and characterized were employed (21). Oligo-metastatic (M14-OL) and poly-metastatic (M14-POL) cell line were generated from three consecutive rounds of *in vivo* selection and passage. They exhibit high (POL cells) and low (OL cells) metastatic colonization efficiency *in vivo*, respectively (21). We show that exosomal crosstalk between metastatic POL and OL cells is a new mechanism of cancer metastasis. High-metastatic melanoma cells (POL) can augment the proliferative colonization capability of the low-metastatic melanoma cells (OL). POL achieves this goal by utilizing its exosomes to deliver functional miRNAs, in this case, miR-411-5p, to the OL cell. Upon entering OL cells, exosomal miR-411-5p enhance metastatic colonization efficiency by activation of the ERK signaling pathway. Moreover, miR-411-5p expression is higher in cancer tissues of other cancer types (colon, lung, rectum) compared with that of respective normal tissues. The clinical relevance of the present finding merits future investigations.

## Materials and Methods

### Cell Culture

GFP-labelled M14 were kindly provided by Dr. Robert Hoffman (University of California San Diego). M14 derivative cell line, OL, POL, OL-NC, OL-miR-411-5p-OE, POL-Rab27a-sh, were grown in DMEM high glucose supplemented (Hycone) containing 10% fetal bovine serum (ExCell Bio, Shanghai, China).

### Animal Experiments

All animal work was conducted in accordance with an approved protocol and carried out in accordance with the institutional animal welfare guidelines of the Chongqing Medical University. Eight-week-old male NOD/SCID mice were obtained from the core facility of Experimental Animal Centre in Chongqing Medical University. Mice were randomized into 2 experimental groups: negative control (OL-NC, n=5), miR-411-5p overexpression (OL-miR-411-5p-OE, n=5). Each mouse was injected with 1×10^6^ tumor cells into caudal vein. All mice were weighted every 2 days and euthanized at Day 25 post tumor cell injection. Sellstrom Z87 fluorescence goggles and an LDP 470 nm fluorescent protein excitation light source was used to examine the metastatic foci at the lung surface of the mice. Microscopic metastatic nodules in the lungs were counted and confirmed by H&E staining.

### Cell Transfection

miR-411-5p mimics, Cy5-labelled miR-411-5p mimics, miR-411-5p inhibitor, *Rab27a* siRNA were obtained from GenePharma (Shanghai, China). Briefly, cells cultured in 6-well culture plates were transfected with each of above reagent *via* Lipofectamine 2000 according to the manufacturer’s handbook. Transfection efficiency was examined after 48h of incubation and the subsequent experiments were performed. The lentivirus particles of miR-411-5p were purchased from Shanghai GeneChem Company. For cell infection, logarithmically grown cells were planted in 24-well plates at a density of 5×10^4^ cells per well. 5ul lentivirus particles was added to each well and then cultured for 3 days. QPCR was performed to confirm the infection efficiency.

### Exosome Isolation

OL and POL cells were let grow in DMEM with 10% fetal bovine serum for 48h. First, the culture medium was transferred to a 50ml centrifuge tube and centrifuged at 800xg for 5min to collect the supernatant. Subsequently, the supernatant was centrifuged at 2000xg for 10min to remove cell debris. afterwards, the supernatant was collected and centrifuged at 10,000xg for 30min followed by ultracentrifugation for 70min at 100,000xg (Optima L-100XP, Beckman Coulter, USA). The pellet was washed with suitable amount of PBS and centrifuged at 100,000xg for 70min. Finally, the precipitation was re-suspended with appropriate PBS and stored at -80°C.

### TEM

Exosomes were fixed with 2.5% glutaraldehyde at 4°C overnight for TEM observation. After washing with deionized water, approximately 20μL of exosomes suspension was loaded on formvar/carbon-coated grids. Exosomes were negatively stained with aqueous phosphotungstic acid for 1min at room temperature and imaged with a transmission electron microscope (JEM-1400PLUS, JEOL).

### Nanoparticle Tracking Analysis

The concentration and size distribution of exosomes were measured by Nanoparticle Tracking Analysis (NTA) according to the manufacturer’s handbook. Exosomes were resuspended in 1 ml PBS and added into the ZetaVIEW S/N 17-310 instrument (PMX, Germany). The size of exosomes was measured based on Brownian motion and the diffusion coefficient and data was analyzed by ZetaView 8.04.02 software.

### miRNA Expression Profiling

RNA extraction, quality testing, library construction, and sequencing were performed at BGI, Wuhan, China.

### Western Blotting

Cells were lysed in 100ul RIPA buffer (Beyotime, Shanghai, China) with 1% PMSF (Beyotime, Shanghai, China). Protein sample was separated by 12% polyacrylamide gels and transferred to polyvinylidene fluoride (PVDF) membranes. The membranes were blocked with 5% milk in Tris-buffered saline for 1h at room temperature, followed by incubation with primary antibodies at 4°C overnight, and subsequent incubation with secondary antibodies at room temperature for 1h. The antibodies used for this study included anti-ERK1/2 (proteintech, 1:800), anti-phosphorylation ERK (proteintech, 1:3000), anti-RAB27A (proteintech, 1:2000), anti-β-actin (proteintech, 1:2000) and goat anti-rabbit IgG (proteintech, 1:2000). Results were analyzed by ImageJ version 6.0. Three independent experiments were done for statistical analysis.

### qRT-PCR Analysis

Total RNA was extracted with TRIzol Reagent (Takara Biotechnology). For mRNA detection, cDNA was synthesized from 2μg of total RNA by using a PrimeScript RT Master Mix (Takara Biotechnology). qRT-PCR analysis was performed using SYBR Green Real-time PCR Master Mix kit (Takara Biotechnology). The relative mRNA expression was calculated using the relative standard curve method (2^-△△CT^). For the reverse transcription of miRNA, Mir-X miRNA First-Strand Synthesis Kit (TaKaRa Biotechnology) was used. The qRT-PCR reaction was performed making using SYBR Green Real-time PCR Master Mix kit (TaKaRa Biotechnology). U6 was used to normalize the results. The sequences were presented in **Table S1**.

### Transwell Migration and Invasion Assays

Transwell inserts containing polycarbonate filters with 8-μm pores were used to perform cell invasion assay with Matrigel (30μl, 1:8 dilution in serum free medium, BD Biosciences) and migration assay without Matrigel. 500μl serum-free medium with 3 × 10^4^ cells was added into the upper chamber and 800μl culture medium with 20% FBS was seeded into the lower chamber. After 24h of incubation, cells on the lower surface of the membrane were fixed with 70% ethanol for 20min at 4°C and stained with crystal violet for 10min. Subsequently, the cells on the upper surface were removed with a cotton swab. Migrated and invaded cells were observed and imaged under light microscopy. Nine random fields from three replicate Transwells were counted for Statistical analysis.

### 6-Well Colony Formation Assay

200 cells were grown in a 6-well plate at 37°C for 1 weeks, followed by fixation in methanol and staining with crystal violet for 10 min before colony counting. Cell colony consisting of > 50 cells were imaged under light microscopy, counted and statistical analysis performed.

### Exosome Labelling and Visualization of Cellular Uptake

POL cells were transfected with Cy5-labelled miR-411-5p mimics and cultured for 3 days. Exosomes were isolated from the culture medium and labelled with the membrane-labelling fluorescent dye PKH26 (Sigma) according to the manufacturer’s instructions. OL cells were seeded into slide and then cocultured with PKH26-labelled exosomes for 6 h, 12 h and 18 h. Cells were fixed in 4% paraformaldehyde and stained with DAPI, and imaged by confocal microscopy (TCS SP2, LEICA).

### Bioinformatics Analysis

TargetScan 7.2 (http://www.targetscan.org/vert_72/) was used to predict target genes of the miR-411-5p. DAVID (https://david.ncifcrf.gov/) and Webgestalt (http://www.webgestalt.org) was used for GO and KEGG pathway analysis. miR-411-5p expression in human cancers was analyzed by TCGA Research Network (http://cancergenome.nih.gov). The survival of melanoma was analyzed by OncoLnc (http://www.oncolnc.org). According to the expression level of miR-411-5p, the first 25% and the last 25% were divided into high expression group and low expression group, respectively.

### Ethical Approval

All animal work was conducted in accordance with an approved protocol and carried out in accordance with the institutional animal welfare guidelines of the Chongqing Medical University.

### Statistical Analysis

All experiments were performed at least three times for statistical analysis. Quantitative results were presented as mean ± SD. Data were analyzed with GraphPad Prism version 5.0 (GraphPad Software Inc., CA, USA) by two-tailed Student’s t-test. P<0.05 was considered statistically significant and was marked with an asterisk. P<0.01 and P<0.001 were considered highly statistically significant and were marked with double asterisks and triple asterisks, respectively.

## Results

### Exosomes of High-Metastatic POL Cells Can Promote Metastatic Colonization of the Low-Metastatic OL Cells

At the distant organ, whether tumor cells with high metastatic potential can enhance the invasiveness of the tumor cells with low metastatic capability has not been determined. If such a possibility exists, it will provide a new mechanism for the development of clinically recognizable metastases and the heterogeneity associated with advanced-stage cancers.

**Figure 1:**
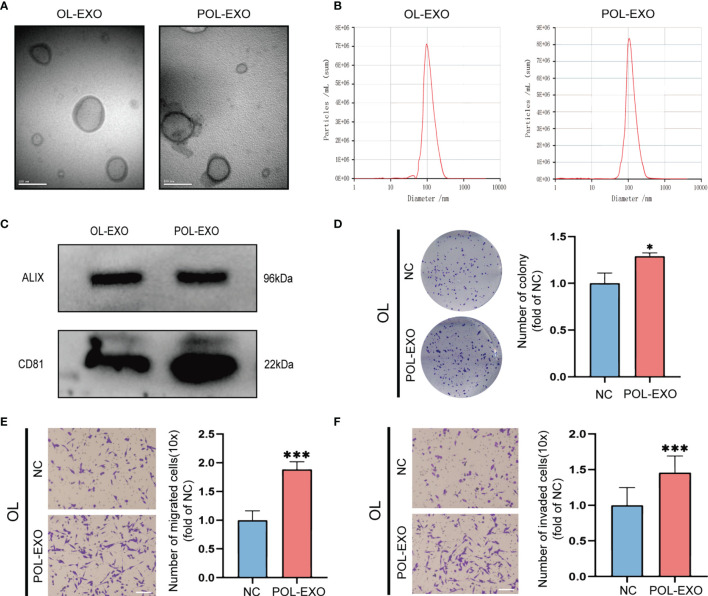
Exosomes derived from POL cells promoted the metastasis of OL cells. **(A)** Representative electron microscopy micrographs of exosomes isolated from OL and POL cells, bar = 100nm. **(B)** NTA analysis to determine the size distribution and number of exosomes. **(C)** WB analysis of exosome markers ALIX and CD81 expression. The effect of exosomes secreted by POL cells (POL-EXO, 40μg) on the proliferation of OL cells was measured using colony formation assays **(D)**, *p < 0.05). Transwell assays without Matrigel or with Matrigel were used to determine the effect of POL-EXO on the migration **(E)**, bar = 60μm, ***p < 0.001) and invasion **(F)**, bar = 60μm, ***p < 0.001) of OL cells, respectively.

Exosomes, as an intercellular communication medium, have been proved to play an important role in tumor metastasis. In this study, the paired metastatic M14-POL and OL cell lines were used to explore whether the tumor cells with high metastatic ability can affect the metastatic ability of tumor cells with low metastatic ability by secreting exosomes. POL and OL cells are different in their invasiveness *in vitro* and metastatic colonization ability *in vivo*. POL cells had stronger ability of migration (**Figure S1A**, p<0.001), invasion (**Figure S1B**, p<0.001) and colony formation (**Figure S1C**, p<0.001) than OL cells *in vitro*. While tail vein injection of POL cells gives arise to extensive multi-organ metastases, injection of OL cells yields limited number of metastatic foci only at the lungs (21). Exosomes of OL and POL cells were isolated and prepared (*Materials and Methods*). The exosomes were characterized for the quality and purity by: transmission electron microscopy (TEM) for visualization of the round or elliptical membranous vesicle morphology (**Figure 1A**); by Nanoparticle Tracking Analysis (NTA) for their size range (average around 100μm, **Figure 1B**); and by Western blot for the expression of exosomal marker proteins ALIX and CD81 (**Figure 1C**) (22). NTA demonstrated that there was little difference in size between exosomes from OL and POL cells. The average diameters of OL and POL exosomes were 96 nm and 107 nm, respectively (**Figure 1B**). We next examined the effect of exosomes derived from POL cells (POL-EXO) on the invasiveness of OL cells *in vitro* by three cell-based functional assays (**Figures 1D–F**). Incubation of OL cells with POL-EXO (40μg) resulted in significant enhancement of the ability of OL cells in colony formation (**Figure 1D**, p<0.5), migration (**Figure 1E**, p<0.001) and invasion (**Figure 1F**, p<0.1001).

**Figure 2:**
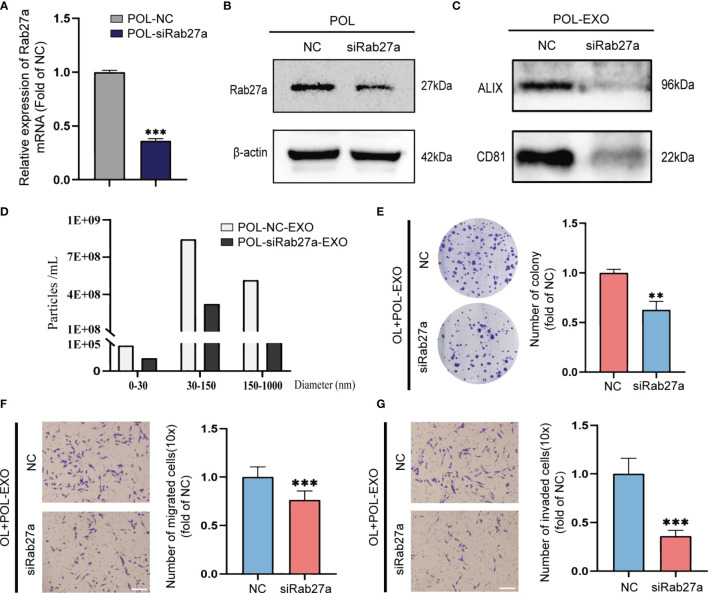
Rab27a knockdown reversed the effect of POL-EXO on the metastasis of OL cells *in vitro*. qPCR measurement of mRNA expression of Rab27a in POL-NC and POL-siRab27a **(A)**, ***p < 0.001). **(B)** WB analysis to confirm effective Rab27a knockdown. **(C)** WB analysis of exosome markers ALIX and CD81 expression in POL-NC-EXO and POL-siRab27a-EXO. **(D)** The concentration of exosomes secreted by POL-NC and POL-siRab27a cells. The effect of exosomes secreted by POL-NC and POL-siRab27a cells on the proliferation of OL cells was measured using colony formation assays **(E)**, **p < 0.01). Transwell assays without Matrigel or with Matrigel were used to determine the effect of exosomes secreted by POL-NC and POL-siRab27a cells on the migration **(F)**, bar = 60μm, ***p < 0.001) and invasion **(G)**, bar = 60μm, ***p < 0.001) of OL cells, respectively.

To confirm that the effect of POL-EXO on *in vitro* invasiveness of OL cells was dependent of the exosomes of POL, we silenced the expression of *Rab27a*, the key regulator of exosome secretion in POL cells. Effective *Rab27a* knockdown was confirmed by qRT-PCR and Western blot, respectively (**Figures 2A, B**). In order to verify the effect of *Rab27a* silencing on exosome secretion, we collected the same volume culture medium of POL-NC and POL-siRab27a cells to extract exosomes. Subsequently, NTA analysis revealed the number of exosomes secreted by POL cells decreased significantly after *Rab27a* silencing (**Figure 2D**). Similarly, the results of western blot showed that the level of exosomal marker ALIX and CD81 in POL-siRab27a group were significantly decreased compared with POL-NC group (**Figure 2C**).

Inhibition of exosome secretion significantly prevented the stimulatory effect of POL-EXO on OL cell invasiveness, as evident by inhibition of colony formation (**Figure 2E**, p<0.01), migration (**Figure 2F**, p<0.001) and invasion (**Figure 2G**, p<0.001). Collectively, these observations demonstrate that POL-EXO can augment the invasiveness of OL cells *in vitro*.

### miR-411-5p Is Significantly Enriched in POL-EXO and Can Be Transferred to OL Cells

**Figure 3:**
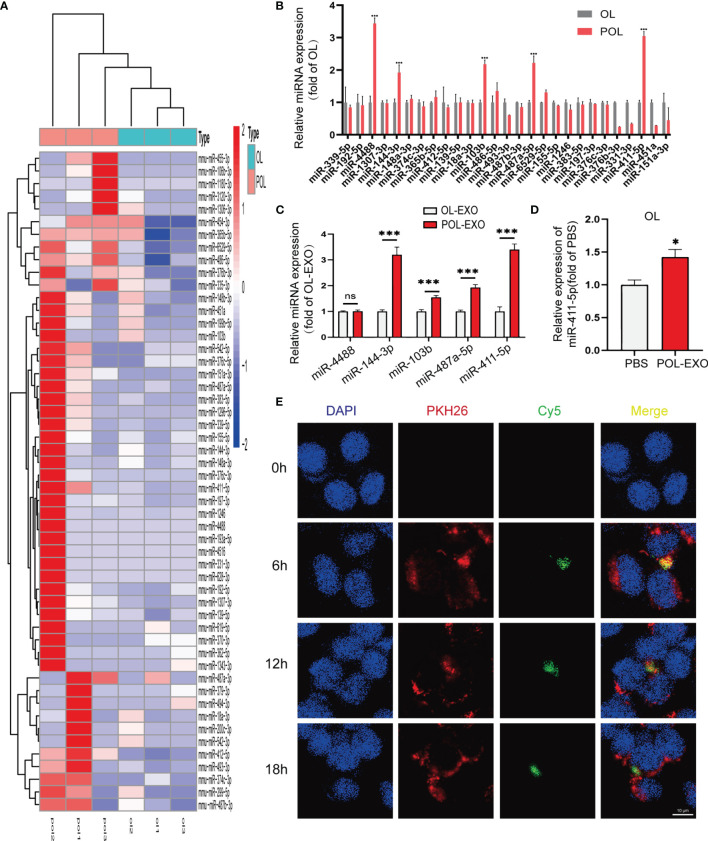
miR-411-5p expression was significantly increased in POL and POL-EXO, and in OL cells upon up-taking of POL-EXO. **(A)** Heat map of miRNA expression profile of POL-EXO and OL-EXO. **(B)** Validation of the expression of 27 up-regulated miRNAs in POL-EXO in OL and POL cells by qRT-PCR. The expression of miR-4488, miR-144-3p, miR-103b, miR-487a-5p and miR-411-5p was further validated in OL-EXO and POL-EXO by qRT-PCR **(C)**, ***p < 0.001). qRT-PCR was used to determine the expression of miR-411-5p in the OL upon co-culture with POL-EXO **(D)**, *p < 0.05). **(E)** Imaging analysis of the time course of OL uptake of exosomes secreted by POL-miR-411-5p-mimics cells, bar = 10μm not significant.

Emerging evidence show that exosomal miRNAs may play an important role in regulating the functions of the recipient cells (23, 24). We speculate that miRNAs contained in exosomes of POL cells are crucial regulators of metastatic function of OL cells. We thus first examined exosomal miRNA expression profile of OL and POL cells by miRNA sequencing (BGI, Wuhan, China, *Materials and Methods*). We found that POL-EXO and OL-EXO could be distinguished by differentially expressed microRNAs (**Figure 3A**). These observations indicate that exosomal microRNA expression is a new feature of cancer cells that have different metastatic capabilities.

Since we are searching for POL-EXO miRNAs that promotes cancer metastasis, we focused on the 27 exosomal miRNAs that had more than 1-fold higher expression in POL-EXO compared to OL-EXO. Results of qRT-PCR validation in POL and OL cells showed that miR-4488, miR-144-3p, miR-103b, miR-487a-5p and miR-411-5p were expressed at significantly high levels in POL cells compared to that of OL cells (**Figure 3B**). We prioritized these five microRNAs for further verification using POL-EXO and OL-EXO. Consistent with the observations in cells (**Figure 3B**), the expression of the four of the five prioritized microRNAs were also expressed at a higher level in POL-EXO than that in OL-EXO (**Figure 3C**, p<0.001). We selected miR-411-5p, which showed stable differential expression and whose functions were largely uncharacterized for further investigation. In addition, miR-411-5p expression was increased in OL cells after treatment with POL-EXO (**Figure 3D**, p<0.5). This result suggests that miR-411-5p was successfully transferred from POL cells to the OL cells *via* exosomes of POL.

To confirm the exosomal route of miR-411-5p delivery from POL cells to OL cells, POL cells were transfected with Cy5-labelled miR-411-5p mimics (green fluorescence) and cultured for 3 days. Exosomes isolated from the POL-miR-411-5p mimics culture medium were labelled with PKH26 (red fluorescence). Afterwards, PKH26-labeled POL-EXO were incubated with OL cells and confocal microscopy imaging was conducted to visualize the time course of POL-EXO (PKH 26-labeled) uptake by OL cells and exosomal miRNA (Cy5-labeled) released within the OL cells. As shown in **Figure 3E**, PKH-labeled POL-EXO entered OL cells and accumulated mostly in the cytosol (**Figure 3E**, 2nd lane, red) as early as after 6h of co-culture. In addition, Cy5-labeled miRNA-411-5p mimic was found inside OL cells (**Figure 3E**, 3rd lane, green). Co-localization of Cy5-miRNA-411-5p mimics with PKH26-POL-EXO (**Figure 3E**, 4^th^ lane, yellow) indicates that miR-411-5p within the POL-EXO can be transferred to and released into the cytosol of the OL cells.

Based on the above observations, we hypothesized that the “high-metastatic POL cells can enhance the metastatic capability of OL cells *via* exosomal transfer of their miR-411-5p to the OL cells”.

### miR-411-5p Promotes the Metastatic Colonization Capability of OL Cells *In Vitro* and *In Vivo*

**Figure 4:**
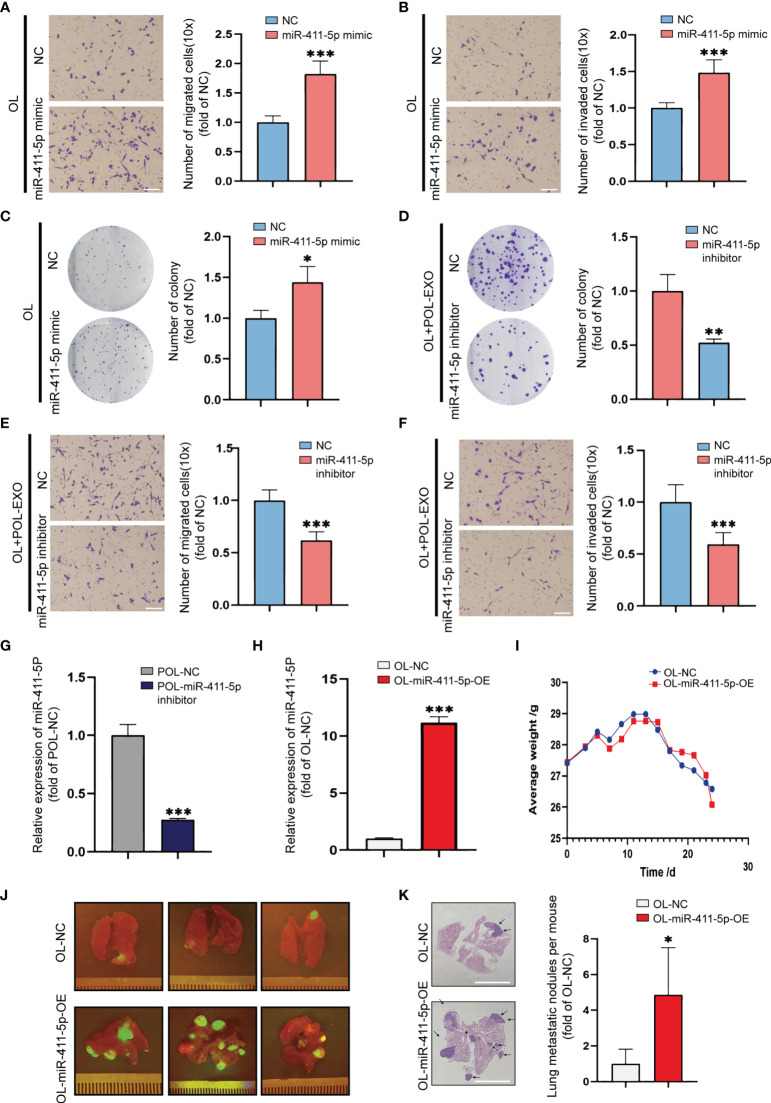
Overexpression of miR-411-5p significantly enhanced the metastatic colonization ability of OL *in vitro* and *in vivo*. Transwell assays without Matrigel or with Matrigel were used to determine the effect of miR-411-5p mimics on the migration **(A)**, bar = 60μm, ***p<0.001) and invasion **(B)**, bar = 60μm, ***p<0.001) of OL cells, respectively. The effect of miR-411-5p mimics on the colony formation ability of OL cells was measured using colony formation assays **(C)**, *p<0.05). The effect of exosomes (40μg) secreted by POL-NC and POL-miR-411-5p inhibitor-treated cells on the colony ability of OL cells was measured using colony formation assays **(D)**, **p<0.01). Transwell assays without Matrigel or with Matrigel were used to determine the effect of exosomes secreted by POL-NC and POL-miR-411-5p inhibitor-treated cells on migration **(E)**, bar = 60μm, ***p<0.001) and invasion **(F)**, bar = 60μm, ***p<0.001), respectively. The expression of miR-411-5p in POL-NC and POL-miR-411-5p inhibitor was measured by qRT-PCR **(G)**, ***p<0.001). The expression of miR-411-5p in OL-NC and OL-miR-411-5p-OE was measured by qRT-PCR **(H)**, ***p<0.001). **(I)** The weight curves of mice injected with OL-NC and OL-miR-411-5p-OE Cells. **(J)** Visualization analysis of the effect of miR-411-5p overexpression on metastatic colonization *in vivo*. H&E staining of metastatic foci in the lungs **(K)**, bar = 5 mm, *p<0.05).

We next studied the effect of miR-411-5p on melanoma metastasis by altering he expression status of miR-411-5p in OL and POL cells, respectively. Overexpression of miR-411-5p in OL cells with transfection of miR-411-5p mimics resulted in significant enhancement of OL cells in migration (**Figure 4A**, p<0.001), invasion (**Figure 4B**, p<0.001) and colony formation (**Figure 4C**, p<0.5). Subsequently, POL cells were transfected with miR-411-5p inhibitor to inhibit the function of miR-411-4p (**Figure 4G**, p<0.001). After transfected with miR-411-5p inhibitor, the colony formation (**Figure S2C**, p<0.001), migration (**Figure S2A**, p<0.001) and invasion (**Figure S2B**, p<0.001) of POL cells all significantly decreased. Compared with POL-NC derived exosomes, the exosomes secreted by POL-miR-411-5p inhibitor cells failed to elicit significant enhancement of OL cells in colony formation (**Figure 4D**, p<0.01), migration (**Figure 4E**, p<0.001) and invasion (**Figure 4F**, p<0.001).

While OL and POL cells are both metastatic, the most pronounced difference of the two models is the metastatic colonization capability *in vivo* (21). As we previously reported, tail vein injection of POL cells yields extensive multi-organ metastases, whereas injection of OL cells produces limited number of metastatic foci only at the lungs (21). To study the effect of miR-411-5p overexpression on OL metastasis *in vivo*, we generated OL-miR411-5p-OE cell line that stably overexpresses miR-411-5p (**Figure 4H**). 1×10^6^ OL-NC and OL-miR-411-5p-OE cells were injected to the immunodeficient NOD/SCID mice by the tail vein to compare metastatic colonization efficiency *in vivo*. Weight changes were monitored (**Figure 4H**) and all mice were sacrificed at day 25 after tumor cell injection. Since OL and POL cells were GFP-labeled, macroscopic metastatic foci formed at the lung surface can be visualize under external fluorescence imaging using Sellstrom Z87 fluorescence goggles and an LDP 470 nm bright blue flashlight as we previously reported (21). There was little difference in body weight between the two groups (**Figure 4I**), and mice inoculated with OL-miR-411-5p-OE cells developed much more GFP-labelled (green fluorescence) macroscopic metastatic foci on the surface of the lungs than that of the OL-NC cells (**Figure 4J**). H&E staining and quantitative analysis also showed miR-411-5p can convert OL cells from oligo-metastatic to poly-metastatic *in vivo*. wed significantly more metastatic foci in the lungs of mice receiving OL-miR-411-5p cells (**Figure 4K**, p<0.5). These observations show that

So far, we have demonstrated that miR-411-5p, transferred from POL-derived exosomes to OL cells, can significantly augment the metastatic colonization capability of OL cells to yield more extensive macroscopic metastases.

### The Pro-Metastatic Activity of miR-411-5p Is Achieved by Activating MAPK/ERK Signaling Pathway

**Figure 5:**
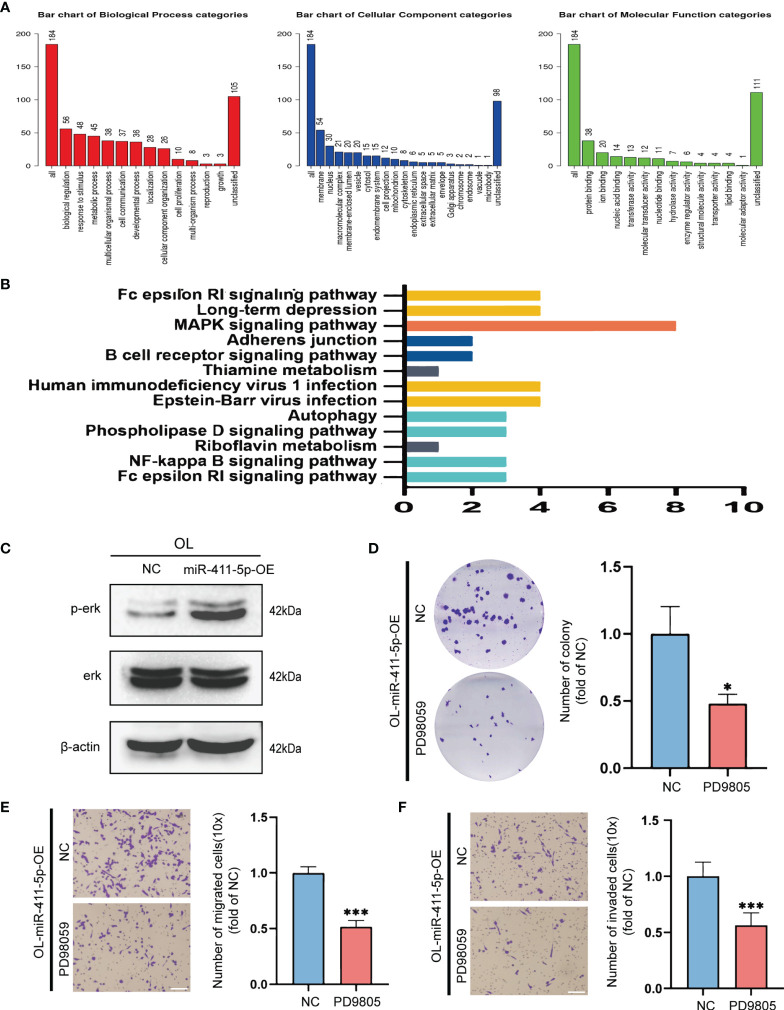
The pro-metastatic activity of miR-411-5p was achieved by activation of the MAPK/ERK signaling pathway. **(A)** GO analysis of the target genes of miR-411-5p. **(B)** KEGG pathway analysis of the target genes of miR-411-5p. **(C)** The protein level of p-Erk and Erk in OL-NC and OL-miR-411-5p mimic. The effect of ERK1/2 inhibitor PD98059 (20μmol/L) treatment on the colony formation of OL-miR-411-5p-OE cells was measured using colony formation assays **(D)**, *p<0.05). Transwell assays without Matrigel or with Matrigel were used to determine the effect of ERK1/2 inhibitor PD98059 (20μmol/L) treatment on the migration **(E)**, bar = 60μm, ***p<0.001) and invasion **(F)**, bar = 60μm, ***p<0.001) of OL-miR-411-5p-OE cells, respectively.

Targetscan (http://www.targetscan.org) was used to perform bioinformatics analysis for predicting the target genes of miR-411-5p. Thereafter, the predicted target genes of miR-411-5p were subjected to GO ontology analysis on Webgestalt (http://www.webgestalt.org) (**Figure 5A**) and KEGG pathway analysis on DAVID (https://david.ncifcrf.gov/) (**Figure 5B**). The “MAPK signaling pathway” was most significantly enriched and was chosen for biological validation.

To validate the predicted relationship between miR-411-5p and MAPK pathway, we assayed phosphorylated ERK (p-ERK) protein levels to determine the activation status of this signaling pathway in OL cells that overexpresseds miR-411-5p (OL-miR-411-5p). Basal level of p-ERK was significantly increased in OL-miR-411-5p-OE group than that in OL-NC group, while the total level of ERK was not changed (**Figure 5C**). This observation indicates that the MAPK/ERK pathway in melanoma cancer cells is subjected to the regulation of miR-411-5p-OE. To determine whether MAPK/ERK activation status will affect the metastatic function of OL cells, MAPK/ERK was inhibited by specific ERK1/2 inhibitor PD98059 (20μmol/L) in OL-miR-411-5p-OE cells, and *in vitro* invasiveness was analyzed. Inactivation of MAPK significantly reversed the stimulatory effect of miR-411-5p overexpression on colony formation (**Figure 5D**, p<0.5), migration (**Figure 5E**, p<0.001) and invasion (**Figure 5F**, p<0.001). Collectively, we demonstrate that the pro-metastatic effect of POL exosomal miR-411-5p on OL cells is achieved by activation of the MAPK/ERK signaling pathway.

**Figure 6:**
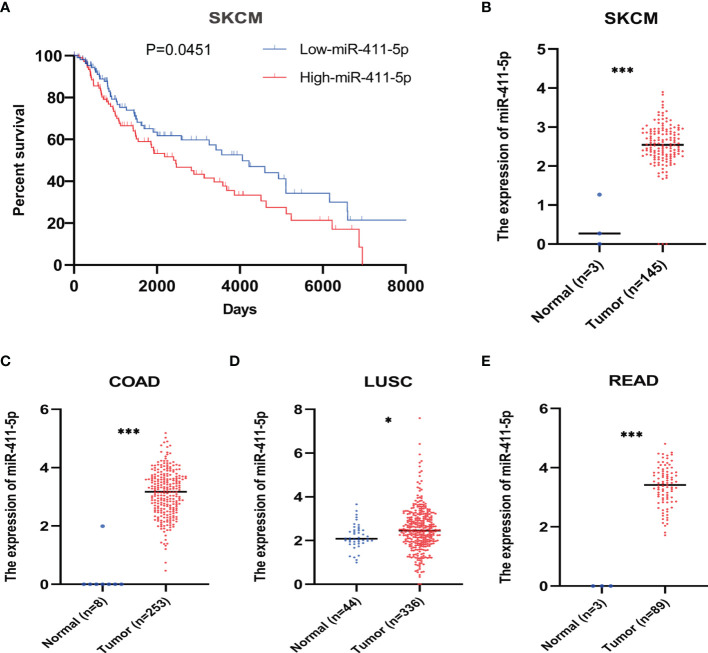
Bioinformatic analysis of miR-411-5p expression in human cancers. **(A)** The overall survival curves of melanoma patients with low expression of miR-411-5p and melanoma patients with high expression of miR-411-5p. The expression of miR-411-5p between normal tissue and tumor in SKCM (Skin Cutaneous Melanoma) **(B)**, ***p < 0.001), COAD (Colon adenocarcinoma) **(C)**, ***p < 0.001), LUSC (Lung squamous cell carcinoma) **(D)**, *p < 0.05) and READ (Rectum adenocarcinoma) **(E)**, ***p < 0.001).

To determine the clinical relevance of miR-411-5p, we downloaded the clinical data associated with miR-411-5p in multiple cancer types from TCGA database for bioinformatics analysis. We found that high expression of miR-411-5p in melanoma patients was significantly associated with poor overall survival (OS) (**Figure 6A**). This is consistent with our biological findings using the clinically relevant melanoma cellular and *in vivo* models. Moreover, miR-411-5p was highly expressed in primary tumors of multiple cancer types compared to the normal tissues (**Figures 6B** **–E**). These results suggest that the exosomal crosstalk mediated by exosomal miR-411-5p between metastatic cancer cells may not be limited to melanoma, and may have a broad implications and relevance for other cancers.

## Discussion

Metastatic melanoma, characterized by high aggressiveness, has a high mortality rate (4). The efficiency of metastasis is mainly limited by the proliferative colonization at the distant organs (5–7). A new microenvironment for tumor growth is required at the stage of colonization and the process is complex and multifactorial. Exosomes, a new class of mediator of cell–cell communication, can transfer biologically active contents (miRNA, mRNA, DNA and proteins) from the donor cell to the target cell (25).

Previous researches on the function of exosomes in metastasis have been primarily focused on tumor cell-derived exosomes in regulating the biological properties of stromal cells (18–20). Exosomal cross-talks between tumor cells and stromal cells are shown to accelerate tumor progression by activation of angiogenesis, immune suppression and the establishment of a niche for metastatic cells (26–29). Whether the extravasated neighboring cancer cells at the involved organ can modify the metastatic capabilities of each other has not been demonstrated before this report. In this paper, we found that exosomes mediate cell-cell interactions between cancer cells with different metastatic abilities can accelerate the metastasis progression.

The present study has made the following novel findings to elucidate a new exosome-based mechanism that underlies the melanoma progression:

***First, exosomes mediated interactions between cancer cells with different metastatic capability, affords more efficient metastatic progression***. Advances in mechanistic understanding of melanoma metastasis have been limited by the lack of clinically relevant metastatic melanoma *in vivo* models. In this study, we employed a paired metastatic models derived from M14 melanoma cell line as we previously characterized (21). OL and POL cells show differences in their metastatic colonization ability *in vivo* and invasiveness *in vitro*. Tail intravenous injection of OL cells produces limited number of metastases at the lungs, whereas injection of POL cells yields extensive multi-organ metastases (21). The differences in proliferative colonization between OL and POL cells *in vivo* provide a platform for us to investigate the hypothesis that interactions between cancer cells may accelerate the progression of melanoma metastasis.

Our findings that exosomes of high-metastatic cancer cells can enhance the metastatic behavior of low-metastatic cells has raised the question - whether the exosomes secreted by low-metastatic cancer cells can weaken the metastatic function of the high-metastatic cancer cells, thereby decelerating metastatic progression. It merits further study.

***Second, Exosomal miR-411-5p from high-metastatic cancer cells (POL) augments the invasiveness of low-metastatic cancer cells (OL), i.e., it is pro-metastatic.*** The involvement of miR-411-5p in oncogenesis and progression has been reported. Both pro- and anti-oncogenic functions of miR-411-5p have been reported and appears to be cancer-type dependent (30, 31). However, the role of miR-411-5p in melanoma, as well as in exosomal crosstalk, have not been investigated prior to this study.

In the present study, we identified the pro-oncogenic activity of miR-411-5p in the context of metastatic colonization and in our cellular models (**Figure 3**). The clinical relevance of this finding was confirmed by bioinformatic analyses of the TCGA database (**Figure 6**). Thus, TCGA analysis results have validated the clinical relevance of the POL and OL models, i.e., the OL and POL models are clinically relevant. miR-411-5p appears to be a poor prognostic marker for melanoma and is pro-metastatic, consistent with our observations in the POL and OL cellular models we employed. Moreover, miR-411-5p expression is higher in cancer tissues of other cancer types (colon, lung, rectum) compared with that of respective normal tissues. This set of findings suggest that the mechanisms we identified here may have broad implications. Validation of the prognostic value of exosomal miR-411-5p with clinical cohorts of melanoma is required.

***Third, the* pro-metastatic activity of miR-411-5p is achieved through activation of the MAPK/ERK signaling pathway.** miR-411-5p is a poorly characterized microRNA and its role in exosomal crosstalk has not been reported. microRNA regulates the expression of target genes at the post-transcriptional level by directly targeting mRNA (32). Thus conventionally, mechanistic understanding of a candidate microRNA is achieved *via* identification of one of its direct gene targets followed by mechanistic illustration of observed effect through the gene function. Such single microRNA-single gene target approach for mechanistic investigation is limited by its over simplification of the complex co-regulations between the transcriptome and the miRNome. Thus, in this study, we performed GO and KEGG pathway analysis of the predicted target genes of miR-411-5p to identify pathways that may be subjected to miR-411-5p regulation. MAPK pathway was most significantly enriched (**Figures 5A** **, B**). In biological validation experiments we show that the activation status of MAPK/ERK is regulated by miR-411-5p expression (**Figure 5C**). Inactivation of MAPK/ERK has reversed the enhancement of *in vitro* invasiveness upon miR-411-5p overexpression (**Figures 5D** **–F**). Target genes enriched in MAPK signaling pathway include *Dups1*, *Spry4*, *Ppm1b* and so on. In particular, DUSP1 is a nuclear mitogen-activated protein kinase (MAPK) phosphatase which can inhibit the phosphorylation of ERK (33). As a negative regulator of MAPK signaling pathway, *Spry4* siRNA can promote p-ERK, p38MAPK, and JNK in PKCα-expressing vector-treated RD cells (34). Therefore, miR-411-5p may activate MAPK/ERK signaling pathway through these target genes. This mode of pathway targeting is more effective than that of targeting a single gene of the same pathway. Thus, pathway targeting could be a mechanism of exosomal miRNA that can effectively modify the metastatic phenotype of the target cells.

In conclusion, we show that exosomal crosstalk between metastatic cancer cells is a new mechanism of cancer metastasis. High-metastatic melanoma cells (POL) can augment the proliferative colonization capability of the low-metastatic melanoma cells (OL). POL achieves this goal by utilizing its exosomes to deliver functional miRNAs, in this case, miR-411-5p, to the OL cell. Upon entering OL cells, exosomal miR-411-5p enhance metastatic colonization efficiency by activation of the ERK signaling pathway. Moreover, prior to the present study, the function of miR-411-5p was poorly characterized. Further, miR-411-5p expression is higher in cancer tissues of other cancer types (colon, lung, rectum) compared with that of respective normal tissues. The clinical relevance of the present finding merits future investigations.

## Data Availability Statement

The data used to support the findings of this study are available from the corresponding authors upon request.

## Ethics Statement

The animal study was reviewed and approved by Biomedical Ethics Committee of Chongqing Medical University.

## Author Contributions

HC and BZ performed the experiments and analyzed data. HC contributed to the writing of this manuscript. XL, QZ, DL, YC, YZ participated in the conduction of this study. HX and JW designed this study, oversaw the execution of this study, and contributed to the writing and revision of this manuscript. All authors contributed to the article and approved the submitted version.

## Funding

This work was supported by the National Natural Science Fund (Grant No. 82073277 and 82173247), the Science and Technology Project Affiliated to the Education Department of Chongqing (Grant No. KJQN202100404), Natural Science Fund of Chongqing (Grant No. cstc2019jcyj-msxmX0868), and Science and Technology Project of Chongqing Yuzhong District (Grant No. 20200110).

## Conflict of Interest

The authors declare that the research was conducted in the absence of any commercial or financial relationships that could be construed as a potential conflict of interest.

## Publisher’s Note

All claims expressed in this article are solely those of the authors and do not necessarily represent those of their affiliated organizations, or those of the publisher, the editors and the reviewers. Any product that may be evaluated in this article, or claim that may be made by its manufacturer, is not guaranteed or endorsed by the publisher.
