# Supplementary figures and images for: High-Metastatic Melanoma Cells Promote the Metastatic Capability of Low-Metastatic Melanoma Cells via Exosomal Transfer of miR-411-5p
Source: Front Oncol. 2022 May 20;12:895164. doi: 10.3389/fonc.2022.895164 (PMC9166236; doi:10.3389/fonc.2022.895164)

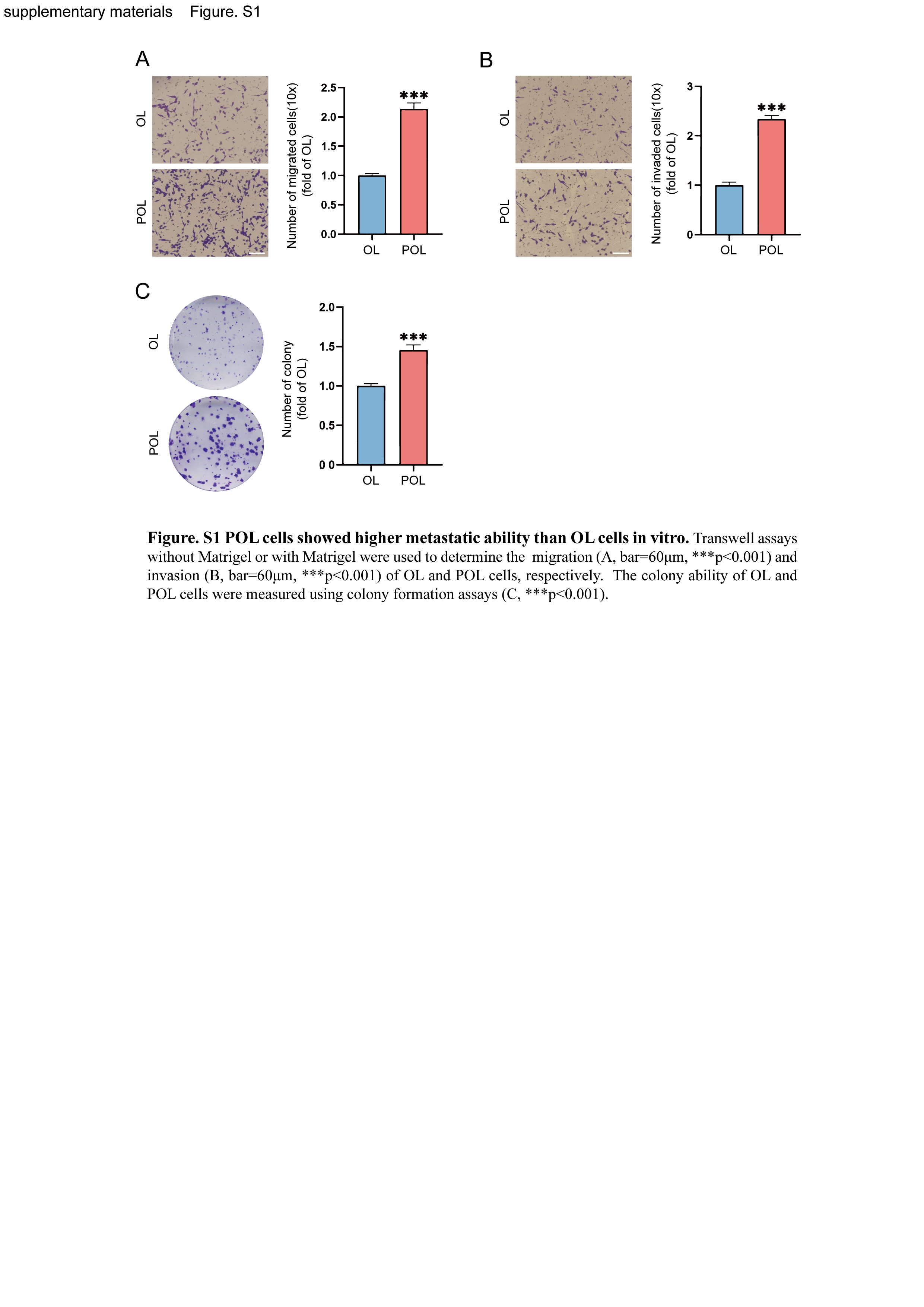

Supplement: Supplementary file 1 [file Image_1.tif]

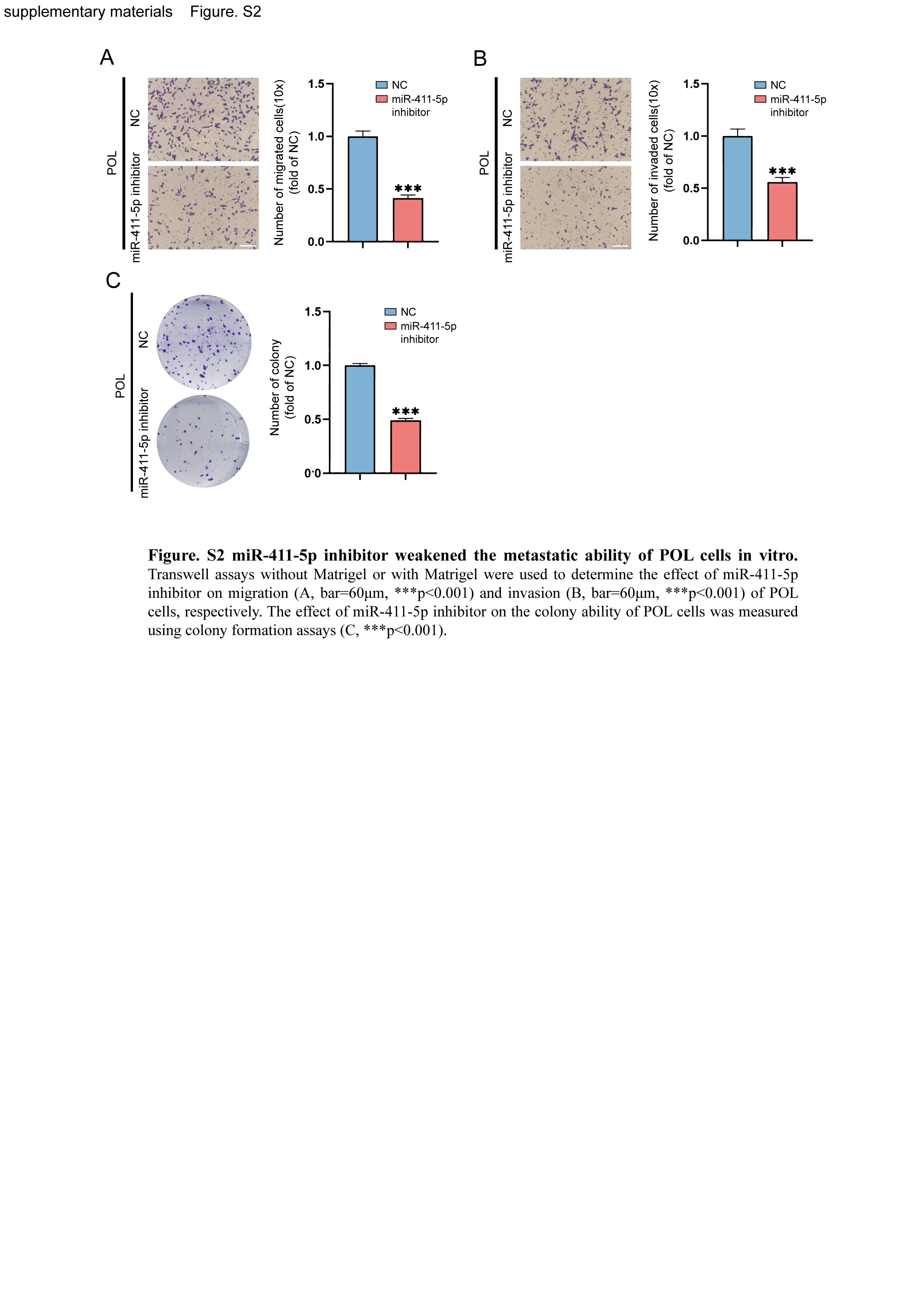

Supplement: Supplementary file 2 [file Image_2.tif]
