# Supplementary material for: High-Metastatic Melanoma Cells Promote the Metastatic Capability of Low-Metastatic Melanoma Cells via Exosomal Transfer of miR-411-5p
Source: Front Oncol. 2022 May 20;12:895164. doi: 10.3389/fonc.2022.895164 (PMC9166236; doi:10.3389/fonc.2022.895164)
Supplement: Supplementary file 3 [file Table_1.pdf]

Table S1. List of primers for qRT-PCR.

gene names	Primers	
	Forward	Reverse
miR-4488	TATATAGGGGGCGGGCTCC	
miR-331-3p	TATAGCCCCTGGGCCTATCCTAGAA	
miR-151a-3p	CGCTAGACTGAAGCTCCTTGAGG	
miR-376c-3p	CGCGAACATAGAGGAAATTCCACGT	
miR-412-5p	CGTGGTCGACCAGTTGGAAAGTAAT	
miR-1246	CGCGAATGGATTTTTGGAGCAGG	
miR-487a-5p	GGTGGTTATCCCTGCTGTGTTCG	
miR-383-5p	CGAGATCAGAAGGTGATTGTGGCT	
miR-1307-3p	TATACTCGGCGTGGCGTCG	
miR-339-5p	TCCCTGTCCTCCAGGAGCTCACG	
miR-197-3p	TTCACCACCTTCTCCACCCAG	
miR-374c-3p	GCGCGCACTTAGCAGGTTGTATTATAT	
miR-493-3p	TGAAGGTCTACTGTGTGCCAGG	
miR-6529-5p	GAGAGATCAGAGGCGCAGAGTG	
miR-411-5p	CCGCTAGTAGACCGTATAGCGTACG	
miR-376b-3p	GCCGCGATCATAGAGGAAAATCCATG	
miR-139-5p	TCTACAGTGCACGTGTCTCCAGT	
miR-155-5p	TACTGCCCTAAGTGCTCCTTCTGG	
miR-486-5p	TCCTGTACTGAGCTGCCCC	
miR-144-3p	CGCCGCGTACAGTATAGATGATGTACT	
miR-18a-3p	TACTGCCCTAAGTGCTCCTTCTGG	
miR-192-5p	CCGCTGACCTATGAATTGACAGCC	
miR-148a-3p	CGCTCAGTGCACTACAGAACTTTGT	
miR-451a	CGCGAAACCGTTACCATTACTGAGTT	
miR-365b-5p	TATAGGGACTTTCAGGGGCAGC	
miR-103b	CTCATAGCCCTGTACAATGCTGCT	
miR-487b-3p	CCGAATCGTACAGGGTCATCCACTT	
Gapdh	ACACCGCTCCATCCGCTTCA	AGGGGCCAT CCACAGTCTTC
Rab27a	AGTGCTGCCAACGGGACAAAC	ACACCGCTCCATCCGCTTCA
U6	CTCGCTTCGGCAGCACA	AACGCTTCACGAATTTGCGT
